# Modeling lung diseases using reversibly immortalized mouse pulmonary alveolar type 2 cells (imPAC2)

**DOI:** 10.1186/s13578-022-00894-4

**Published:** 2022-09-22

**Authors:** Linghuan Zhang, Wenping Luo, Jiang Liu, Maozhu Xu, Qi Peng, Wenjing Zou, Jingyi You, Yi Shu, Piao Zhao, William Wagstaff, Guozhi Zhao, Kevin Qin, Rex C. Haydon, Hue H. Luu, Russell R. Reid, Yang Bi, Tianyu Zhao, Tong-Chuan He, Zhou Fu

**Affiliations:** 1 Stem Cell Biology and Therapy Laboratory, Ministry of Education Key Laboratory of Child Development and Disorders, and the Department of Respiratory Diseases, The Children’s Hospital of Chongqing Medical University, Chongqing, 400014 China; 2 Molecular Oncology Laboratory, Department of Orthopaedic Surgery and Rehabilitation Medicine, The University of Chicago Medical Center, 5841 South Maryland Avenue, MC3079, Chicago, IL 60637 USA; 3 Laboratory Animal Center, Southwest University, Chongqing, 400715 China; 4 University-Town Hospital, Chongqing Medical University, Chongqing, 401331 China; 5 Departments of Orthopaedic Surgery and Urology, The First Affiliated Hospital of Chongqing Medical University, Chongqing, 400046 China; 6 Rosalind Franklin University of Medicine, North Chicago, IL 60064 USA; 7 Department of Surgery, The University of Chicago Medical Center, Chicago, IL 60637 USA; 8 Laboratory of Craniofacial Suture Biology and Development, Department of Surgery Section of Plastic Surgery, The University of Chicago Medical Center, Chicago, IL 60637 USA; 9 Chongqing Key Laboratory of Oral Diseases and Biomedical Sciences, the Stomatological Hospital of Chongqing Medical University, Chongqing, 401147 China

**Keywords:** Alveolar type 2 cells (AT2), Pulmonary alveolar epithelium, Immortalization, Alveolar organoids, Lung progenitors, Pulmonary fibrosis, Lung tumorigenesis

## Abstract

**Background:**

A healthy alveolar epithelium is critical to the gas exchange function of the lungs. As the major cell type of alveolar epithelium, alveolar type 2 (AT2) cells play a critical role in maintaining pulmonary homeostasis by serving as alveolar progenitors during lung injury, inflammation, and repair. Dysregulation of AT2 cells may lead to the development of acute and chronic lung diseases and cancer. The lack of clinically relevant AT2 cell models hampers our ability to understand pulmonary diseases. Here, we sought to establish reversibly immortalized mouse pulmonary alveolar type 2 cells (imPAC2) and investigate their potential in forming alveolar organoids to model pulmonary diseases.

**Methods:**

Primary mouse pulmonary alveolar cells (mPACs) were isolated and immortalized with a retroviral expression of SV40 Large T antigen (LTA). Cell proliferation and survival was assessed by crystal violet staining and WST-1 assays. Marker gene expression was assessed by qPCR, Western blotting, and/or immunostaining. Alveolar organoids were generated by using matrigel. Ad-TGF-β1 was used to transiently express TGF-β1. Stable silencing β-catenin or overexpression of mutant KRAS and TP53 was accomplished by using retroviral vectors. Subcutaneous cell implantations were carried out in athymic nude mice. The retrieved tissue masses were subjected to H & E histologic evaluation.

**Results:**

We immortalized primary mPACs with SV40 LTA to yield the imPACs that were non-tumorigenic and maintained long-term proliferative activity that was reversible by FLP-mediated removal of SV40 LTA. The EpCAM^+^ AT2-enriched subpopulation (i.e., imPAC2) was sorted out from the imPACs, and was shown to express AT2 markers and form alveolar organoids. Functionally, silencing β-catenin decreased the expression of AT2 markers in imPAC2 cells, while TGF-β1 induced fibrosis-like response by regulating the expression of epithelial-mesenchymal transition markers in the imPAC2 cells. Lastly, concurrent expression of oncogenic *KRAS* and mutant *TP53* rendered the imPAC2 cells a tumor-like phenotype and activated lung cancer-associated pathways. Collectively, our results suggest that the imPAC2 cells may faithfully represent AT2 populations that can be further explored to model pulmonary diseases.

**Supplementary Information:**

The online version contains supplementary material available at 10.1186/s13578-022-00894-4.

## Background

The primary function of the lungs is oxygen/CO2 gas exchange, which occurs in the alveoli arranged by acini in the lung parenchyma regions [1, 2]. The pulmonary alveolar epithelium also prevents pathogens and fluids from entering airspaces [1, 3]. Thus, a functional alveolar-capillary barrier is critical for maintaining efficient gas exchange, preventing vascular leakage, and facilitating fluid clearance [4]. The alveolar-capillary barrier consists of three major components: the alveolar epithelium containing the squamous alveolar type 1 (AT1) cells and surfactant-producing cuboidal AT2 cells, the alveolar capillary endothelium, and the alveolar basement membrane of sparse connective tissue interstitium [1, 2, 4]. Intercellular junctional complexes such as cadherin-expressing adherens junctions and claudin-rich tight junctions play an essential role in regulating the permeability across the alveolar epithelium and endothelium. Insufficient generation of alveoli causes various neonatal and infant diseases including bronchopulmonary dysplasia (BPD)[4, 5]. Damage of the alveolar region and/or destabilization of epithelial or endothelial junctions are frequently key features seen in acute lung injury, acute respiratory failure in pneumonia, acute respiratory distress syndrome (ARDS), chronic adult lung diseases such as chronic obstructive pulmonary disease (COPD) and idiopathic pulmonary fibrosis (IPF), and microbial infections such as SARS-CoV-2 infection in COVID-19 [4–6].

AT2 cells account for approximately 60% of alveolar epithelial cells, and play a critical role in maintaining pulmonary homeostasis by producing surface tension-reducing surfactants and regulating local immune responses [3, 6–8]. Increasing evidence indicates that AT2 cells are highly heterogeneous and serve as alveolar progenitors that can give rise to both AT1 and AT2 populations during lung injury, inflammation, and repair [5, 6, 9, 10]. It has been shown that Wnt signaling is critical in maintaining AT2 stemness and regulating the differentiation of AT2 progenitors to AT1 cells [11–13]. Abnormal functions of AT2 cells have been implicated in the pathogenesis of many serious pulmonary diseases including pulmonary fibrosis, ARDS, chronic obstructive pulmonary disease, and lung cancer [1, 2, 5, 6].

Given the functional importance of alveolar epithelial cells, in particular the AT2 cells, numerous efforts have been devoted to isolate primary lung epithelial cells from humans, rats, and mice [14–16]. However, the limited life span of the primary alveolar epithelial cells prevents them from widespread applications in studying lung diseases. Several commonly used lung epithelial cell lines are human lung cancer (e.g., A549 and H441), mouse SV40 Large T transgenic lung tumors (e.g., MLE12 and MLE15), and spontaneously immortalized Golden hamster (e.g., M3E3/C3) cell lines, to name a few [17], although many of these lines do not always recapitulate the morphological, functional, and/or phenotype of the AT2 cells. Interestingly, it has been reported that lung organoids can be generated from human pluripotent stem cells (hPSCs) although the process is finicky and time consuming [18, 19]. Despite the pivotal role of alveoli and AT2 cells in lung function and disease, the lack of clinically relevant cell models hampers our understanding about lung biology and pathogenesis of pulmonary diseases.

In this study, we sought to establish the reversibly immortalized mouse pulmonary alveolar type 2 cells (imPAC2) and investigate their potential in forming alveolar organoids to model pulmonary diseases. Experimentally, primary mouse pulmonary alveolar cells (mPACs) were immortalized with SV40 Large T antigen (LTA) that is flanked with FRT sites, yielding the pooled imPACs that maintain long-term proliferative activity but are not tumorigenic. The immortalization phenotype of imPACs was reversible by FLP-mediated removal of SV40 LTA. AT2-enriched subpopulation (imPAC2) was obtained by FACS sorting for the EpCAM^+^ cells from the imPACs, and was shown to express AT2 markers and form alveolar organoids. Silencing β-catenin significantly decreased the expression of AT2 markers in the imPAC2 cells, while TGF-β1 induced alveolar fibrosis-like response by regulating the expression of epithelial-mesenchymal transition (EMT) markers in the imPAC2 cells. Lastly, concurrent expression of oncogenic *KRAS* and mutant *TP53* in the imPAC2 cells induced a tumor-like phenotype and activated lung cancer-associated pathways. Collectively, our results strongly suggest that the imPAC2 cells may faithfully represent AT2 populations so they can be exploited to form AT2-based alveolar organoids for modeling pulmonary diseases.

## Methods

### Cell culture and chemicals

Human HEK293 and its derivative lines 293pTP and RAPA were described previously [20, 21]. Human lung cancer line PC-9 was obtained from American Type Culture Collection (ATCC, Manassas, VA). All cell lines were cultured in complete DMEM containing 10% fetal bovine serum (FBS, Invitrogen, Carlsbad, CA) with penicillin/streptomycin, and maintained at 37 °C in 5% CO_2_ as described [20, 22–24]. Unless indicated otherwise, all chemicals were purchased from Sigma-Aldrich (St. Louis, MO) or Thermo Fisher Scientific (Waltham, MA).

### Isolation and culture of primary mouse pulmonary alveolar cells (mPACs)

The use and care of animals were approved by the Institutional Animal Care and Use Committee of The University of Chicago. Experimentally, freshly prepared lung lobe tissues were retrieved from multiple CD1 newborn mice, washed with ice-cold sterile PBS, and minced into small pieces of approximately 1.0mm^3^. The minced pulmonary lobule-rich tissue bits were plated into 6-well cell culture plates and maintained in DMEM containing 10% FBS and penicillin/streptomycin. At days after culturing, lung tissues were removed, and the attached lung alveolar cells were passaged, pooled and designated as primary mouse pulmonary alveolar cells (mPACs). The mPACs within two passages were used for the subsequent experiments.

### Establishment of reversibly immortalized mouse pulmonary alveolar cells (imPACs) and sorted alveolar type 2 cells (imPAC2)

We immortalized mPACs with the retroviral vector SSR#41, which expresses the SV40 LTA flanked with FRT sites as previously described [25–36]. Briefly, subconfluent HEK293 cells were co-transfected with SSR#41, pCL-Ampho and pCMV-VSVG to package retrovirus as described previously [37–41]. The vial supernatants were collected, cleared of cell debris, and used to infect subconfluent primary mPACs. The infected primary mPACs were selected with hygromycin B (at final concentration of 3 µg/mL), and subsequently yielded the immortalized mouse mPAC cell pool, designated as imPACs.

After three to five passages, the imPACs were subjected to magnetic cell sorting (MACS, Miltenyi Biotec) using EpCAM^+^ beads to enrich and isolate pulmonary alveolar type 2 (AT2) cells, as previously described [42]. After being validated for SP-C positivity by FACS analysis and qPCR and immunostaining assays of other AT2 markers, the isolated AT2 cells were designated as imPAC2 cells, which were used for further experimentation.

### Construction and amplification of recombinant adenoviruses expressing TGFβ1, Flippase recombinase (FLP), GFP and RFP

Recombinant adenoviruses were constructed using the AdEasy technology and recently developed OSCA system as previously described [43–46]. Briefly, coding regions of FLP and mouse TGFβ1 were PCR amplified and cloned into an adenoviral shuttle vector, followed by homologous recombination with the adenoviral backbone vector in BJ5183 bacterial cells. The resultant adenovirus plasmids were used to generate adenoviruses in 293pTP and RAPA packaging cells as described [20, 21], leading to the generation of recombinant adenoviruses Ad-TGFβ1, Ad-FLP. The Ad-FLP co-expresses GFP marker, whereas the Ad-TGFβ1 co-expresses RFP marker. Analogous adenovirus expressing RFP (Ad-RFP) or GFP (Ad-GFP) alone was constructed by using the recently developed OSCA system [46].

### Construction of the imPAC2-simBC line using the multiplex siRNA expression system to silence mouse β-catenin expression in imPAC2 cells

In order to effectively silence β-catenin expression in imPAC2 cells, we took advantage of our recently-developed multiplex siRNA expression systems [39, 47, 48]. Specifically, we designed three pairs of siRNAs that target the coding region of mouse β-catenin and constructed a multiplex siRNA vector in the retroviral pSEB361-BSG vector using the BSG versatile shotgun cloning system as previously described [48], resulting in the pSEB361-simBC vector. Retrovirus packaging was carried out as described [49]. The retrovirus supernatants were used to infect subconfluent imPAC2 cells, which were subsequently subjected to blasticidin S selection (3 µg/mL), yielding a stable pool of imPAC2-simBC cells. The DNA sequences for the three siRNAs targeting mouse β-catenin are listed in Additional file 1: Table S1.

### Construction of the imPAC2-KRAS/TP53R273H line that stably expressed both oncogenic *KRAS* and mutant *TP53*

Plasmids containing the full-length coding regions of human KRAS-G12C and human TP53-R273H mutations were kindly provided by Dr. Lin Zhang of the University of Pittsburgh Cancer Institute. The coding regions of human KRAS-G12C and TP53-R273H were amplified with high fidelity PCR, and individually subcloned into a homemade retroviral vector pSEBI, resulting in pSEBI-KRAS and pSEBI-TP53R273H, respectively. All PCR amplified sequences and the cloning junctions were verified by DNA sequencing. The retroviral vectors were co-transfected into HEK293 cells with pCL-Ampho and pCMV-VSVG to package retrovirus. The retrovirus supernatants were used to infect the imPAC2 cells, which were subsequently subjected to blasticidin S selection (3 µg/mL), yielding a stable pool of imPAC2-KRAS/TP53R273H cells.

### RNA isolation and touchdown-quantitative PCR analysis (TqPCR)

Subconfluent cells were subjected to various treatments and total RNA was isolated at the indicated time points using TRIZOL reagent. The reverse transcription was performed by using random hexamer and M-MuLV (New England Biolabs, Ipswich, MA). The RT products were used, and qPCR primers were designed by using the Primer3 Plus. The TqPCR analysis was carried out as described previously [50–53]. All transcripts were normalized by using *Gapdh* as a reference gene [54–57]. The qPCR primers are listed in Additional file 1: Table S1.

### Western blotting analysis of the AT2 markers

Western blotting analysis was carried out as described [51, 58, 59]. Briefly, exponentially growing imPAC2 cells were seeded in 60 mm dishes and cultured for 48 h. The supernatants were collected and dissolved in RIPA lysis buffer containing protease inhibitors, while the cells were directly lysed with RIPA lysis buffer containing protease inhibitors. The samples were subjected to SDS-PAGE and transferred to PVD membranes (Millipore). The membranes were blocked in 5% fat-free skimmed milk for 1 h, then incubated with antibodies against SftpA, SftpB, SftpD (Novus Biologicals, Shanghai, China), SftpC and β-actin (Abcam, Shanghai, China, 1:500) at 4 °C overnight, and subsequently incubated with respective secondary antibodies. The proteins of interest were visualized by using the ECL Western Blotting Substrate Kit (Abcam, Shanghai, China). β-Actin (from cell lysate) was used as an internal control for both cell lysate and supernatant samples.

### Immunofluorescence staining

Immunofluorescence staining was conducted as described [30, 60, 61]. Briefly, subconfluent imPAC2, imPAC2 organoids, and frozen sections of imPAC2 and imPAC2-simBC implants, were fixed with paraformaldehyde and permeabilized with 0.1% Triton X-100. The cells and sections were washed with PBS, blocked with goat serum, and incubated with primary antibodies against SftpA, SftpB, SftpD (Novus Biologicals), and/or SftpC (Abcam), followed by detection with fluorescence probes labelled secondary antibodies. Negative controls were stained without primary antibodies. Cell nuclei were counterstained with DAPI. Fluorescence images were recorded with fluorescence microscope or confocal microscopy.

### Xenogen bioluminescence imaging and in vivo tumorigenicity assay

The human lung cancer cell line PC9 and imPACs were stably tagged with a retroviral vector expressing firefly luciferase (FLuc), resulting in PC9-FLuc and imPAC-FLuc lines, respectively. Exponentially growing PC9-FLuc and imPAC-FLuc cells were collected and resuspended in sterile PBS at 2 × 10^7^ cells/mL. The cells were injected subcutaneously into the flanks of athymic nude mice (2 × 10^6^ cells per injection, 6 injection sites per animal, four male mice per group, 6-week old, ENVIGO Harlan Laboratories, USA). The mice were imaged with the Xenogen IVIS 200 system at day 3 and day 10. The subcutaneous injection sites were monitored for over 4 weeks and the imaging results were quantitatively analyzed with the Xenogen’s Living Image software as previously reported [56, 62–65].

### Organoid formation

Subconfluent imPACs and imPAC2 cells were infected with Ad-GFP for 16 h. The cells were collected and seeded in ultralow adhesive 24-well plates at 5,000 cells/well in 100 μL per well of Matrigel (BD Biosciences). The whole process was performed on ice and followed by an incubation step for 30 min at 37 °C in 5% CO_2_ incubator for Matrigel polymerization. Complete DMEM was then added carefully to each well (1000 μL per well). The medium was exchanged every three days, and live cell imaging was performed under a fluorescence microscope at the indicated time points. The spheroid organoids were harvested at 14 and 21 days for immunofluorescence staining as described [27, 66].

### Subcutaneous imPAC2 cell implantation

The cell implantation experiments were carried out as described [67–71]. In order to sustain long-term cell survival, we used the previously characterized biocompatible and thermoresponsive PPCNg, poly(polyethyleneglycol citrate-co–N-isopropylacrylamide) (PPCN) mixed with gelatin, as a scaffold and delivery vehicle for imPAC2 implantation as previously described [29, 36, 52, 72–74]. For the cell implantation experiments, exponentially growing imPAC2, imPAC2-simBC, or imPAC2-KRAS/TP53R273H (10^6^ cells per injection) were collected and resuspended in 80µL of sterile ice-cold PPCNg mix (40µL 1% PPCN in PBS + 40 µL 0.2% gelatin). The cell/PPCNg mix was subcutaneously injected into the flanks of athymic nude mice (6–8 week old, male, n = 4/group, 4 injection sites per mouse, 10^6^ cells per injection). Animals were fed ad libitum and sacrificed at 30 days after implantation. The masses at the implantation sites were isolated for qPCR, histological and immunostaining analyses.

For the TGFβ1 stimulation study, subconfluent imPAC2 cells were infected with Ad-TGFβ1 or Ad-RFP for 16 h, and then collected and resuspended in sterile ice-cold PPCNg mix as described above. The infected cell/PPCNg mix was subcutaneously injected into the flanks of athymic nude mice (6–8 week old, male, n = 4/group, 4 injection sites per mouse, 10^6^ cells per injection). Animals were fed ad libitum and sacrificed at 30 days after implantation. The retrieved samples were subjected to qPCR and histological analyses.

### Statistical analysis

All quantitative assays were performed in triplicate. The quantitative data are presented as mean ± standard deviation (SD). SPSS software (version 17.0) (one-way analysis of variance) was used to determine statistical significance. Statistical significance was set at *P* < 0.05.

## Results

### Establishment of reversibly immortalized mouse pulmonary alveolar cells (imPACs) with long-term proliferative activity

**Fig. 1:**
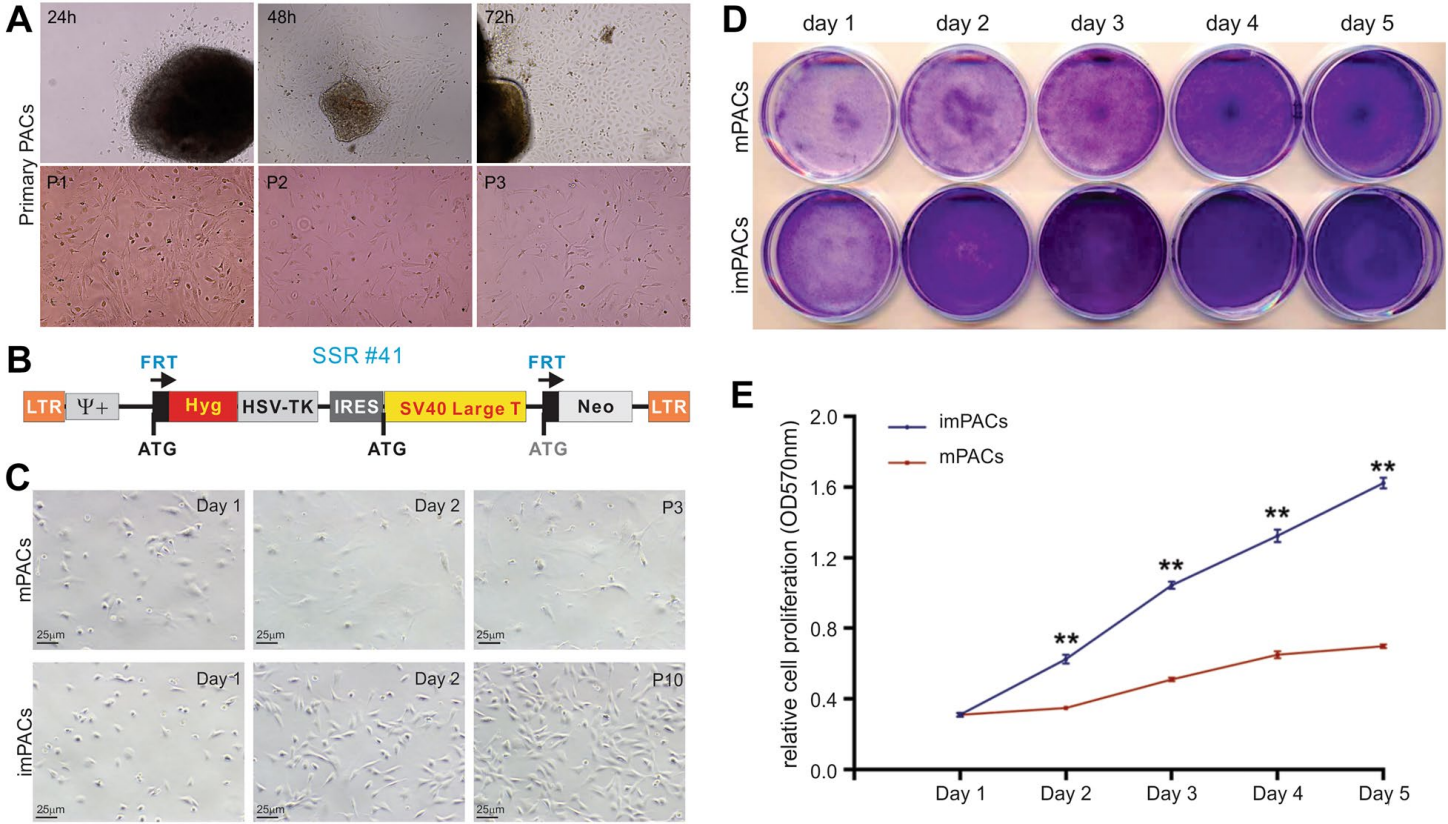
Establishment of the reversibly immortalized mouse pulmonary alveolar cells (imPACs). **A** Isolation of primary mouse pulmonary alveolar cells (mPACs) from the alveoli of newborn CD1 mice. The primary PACs cells migrating out of the minced lung tissues barely survived passage 4.**B** A schematic representation of the reversible immortalization retroviral vector SSR #41. This vector contains a cistronic expression cassette for the expression of both hygromycin resistance and SV40 Large T-antigen (SV40 Large T, or SV40 LTA flanked with the FRT sites, which can be removed by Flippase recombinase (FLP). **C** Establishment of imPACs. Subconfluent mPACs were infected with the SSR#41retrovirus supernatant and selected with hygromycin B, leading to the establishment of imPACs. The mPACs and imPACs were seeded at the same density, passaged and photographed at the indicated time points. Representative images are shown. **D** Cell proliferation and viability assay. Subconfluent mPACs and imPACs were seeded at the same density and subjected to crystal violet staining at the indicated time points. Representative images are shown. **E** The crystal violet stained cells were dissolved for quantitative determination at A570 nm. “**” p < 0.01 compared that of the mPACs

We isolated primary mouse pulmonary alveolar cells (mPACs) from the lung lobes of CD1 newborn pups; and the isolated mPACs were cultured for up to three passages (Fig. 1A). The freshly isolated mPACs were then immortalized with a retroviral vector SSR#41, which expresses the SV40 LTA and hygromycin resistance gene (Fig. 1B), and has allowed us to immortalize a broad range of mouse and human primary cells [25–36]. The infected mPACs were subjected to hygromycin selection, and the survived cells were continuously cultured for more than 10 passages, and then pooled and designated as imPACs.

Compared with the primary mPACs, the imPACs grew rather quickly and proliferated well after 10 passages (Fig. 1C). We further compared the cell proliferation and viability by crystal violet staining, and found that imPACs grew faster than the mPACs under the same condition (Fig. 1D), which was further confirmed by quantitative analysis of the crystal violet staining assay (Fig. 1E). In fact, the imPACs have been successfully passaged for more than 50 generations to date (data not shown). Collectively, these results indicate that the imPACs exhibit high proliferative activity and can be cultured for long-term.

### FLP-mediated removal of SV40 LTA significantly decreases the proliferative activity of imPACs and the imPACs are non-tumorigenic in vivo

**Fig. 2:**
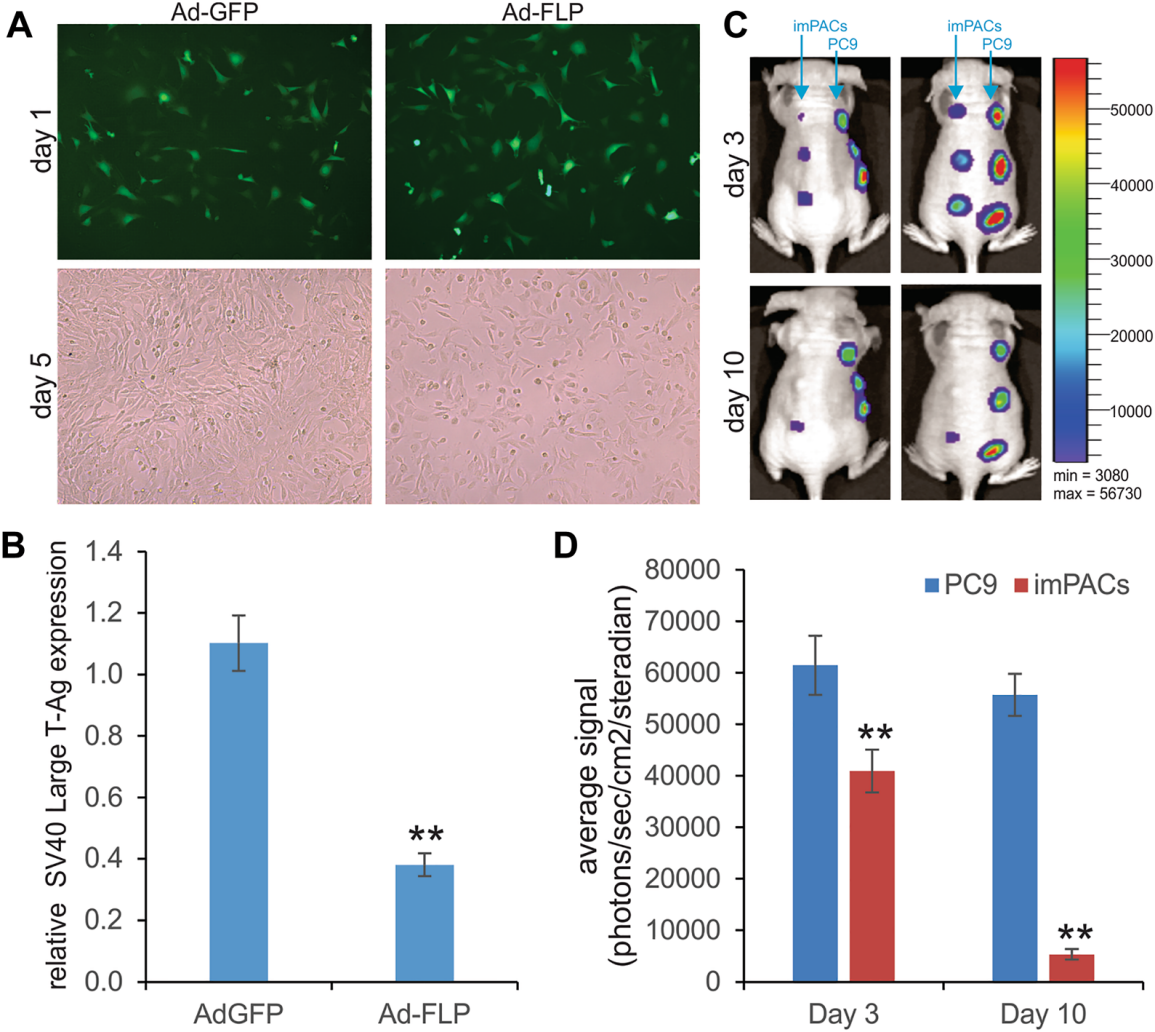
FLP recombinase-mediated removal of SV40 LTA effectively reverses the cell proliferative activity of imPACs, while imPACs are non-tumorigenic in vivo. **A** & **B** Subconfluent imPACs were infected with Ad-GFP or Ad-FLP with high efficiency as shown on 24 h (1 day) post infection (**A**). Total RNA was isolated at 48 h after infection and subjected to RT-qPCR analysis to determine SV40 LTA expression using SV40 LTA-specific primers (**B**). ** p < 0.01 compared with that of the Ad-GFP infection group. **C** & **D** The imPACs are not tumorigenic in vivo. The imPAC and human lung cancer PC9 cells were stably tagged with firefly luciferase (FLuc) and subcutaneously injected into athymic nude mice as described in Methods. The mice were subjected to whole body optic bioluminescence imaging at the indicated time points (**C**). Representative images are shown. The acquired data were quantitatively analyze using the Living Image software (**D**). ** p < 0.01, compared with that of the PC9 cell injection group

In order to test whether the immortalization phenotype can be reversed by the removal of the SV40 LTA, we infected the imPACs with Ad-FLP or AD-GFP control virus with the same infection efficiency, and found that the Ad-FLP infected imPACs grew significantly slower than the Ad-GFP infected cells (Fig. 2A). The qPCR analysis confirmed that Ad-FLP infection reduced more than 65% of SV40 LTA expression, compared with the control group (Fig. 2B). These results strongly indicate that the immortalization phenotype is reversible in the imPACs.

Since exogenous overexpression of SV40 LTA may increase the risk of tumorigenesis [37], we tested whether the imPACs were able to form tumor-like masses in vivo. We stably tagged the imPACs and a positive control line, human lung cancer cell line PC9, with the firefly luciferase FLuc, yielding imPAC-FLuc and PC9-FLuc, respectively. When both cell lines were subcutaneously injected into athymic nude mice, whole body live bioluminescence imaging analysis indicated that the bioluminescence signals in the imPAC-FLuc group were noticeably lower than that of the PC9-FLuc group at as early as day 3; and the signals almost disappeared in the imPAC-FLuc group at day 10, while the PC9-FLuc group maintained strong signals at both time points (Fig. 2C). Quantitative analysis revealed the drastic differences in bioluminescence signals between the imPAC-FLuc and PC9-FLuc cells (p < 0.01) (Fig. 2D). We continued to monitor the injected animals for up to 30 days, and no masses were observed at the injection sites of the imPAC-FLuc group (data not shown). In fact, the subcutaneous bumps at the injection sites in the imPAC-FLuc group completely disappeared at 10–14 days after injection. Thus, consistent with our previous experience with SV40 Large T antigen immortalized cells [25–36], the immortalized imPACs are not tumorigenic in athymic nude mice.

### The imPACs express most of the pulmonary alveolar cell markers and contain the pulmonary alveolar type 2 (imPAC2) cells

We next analyzed whether the imPACs expressed the biomarkers for pulmonary alveolar type 1 (AT1) and type 2 (AT2) cells, which are considered as progenitors of pulmonary alveoli. TqPCR analysis revealed that, while the expression levels of *Pdpn, Ager, SftpC,* and *Muc1* were significantly higher in the imPACs than that in the primary mPACs (Additional file 1: Figure S1A & S1B), the expression levels of other marker genes including *Aqp5*, *Ctsh*, *Nkx2.1*, *SftpB*, *SftpD*, *Abca3*, *Lamp1*, and *Lamp2* were similar in both imPACs and mPACs (p > 0.05) (Additional file 1: Figure S1A to S1C). These results strongly indicate that the imPACs express most of pulmonary alveolar cell markers.

**Fig. 3:**
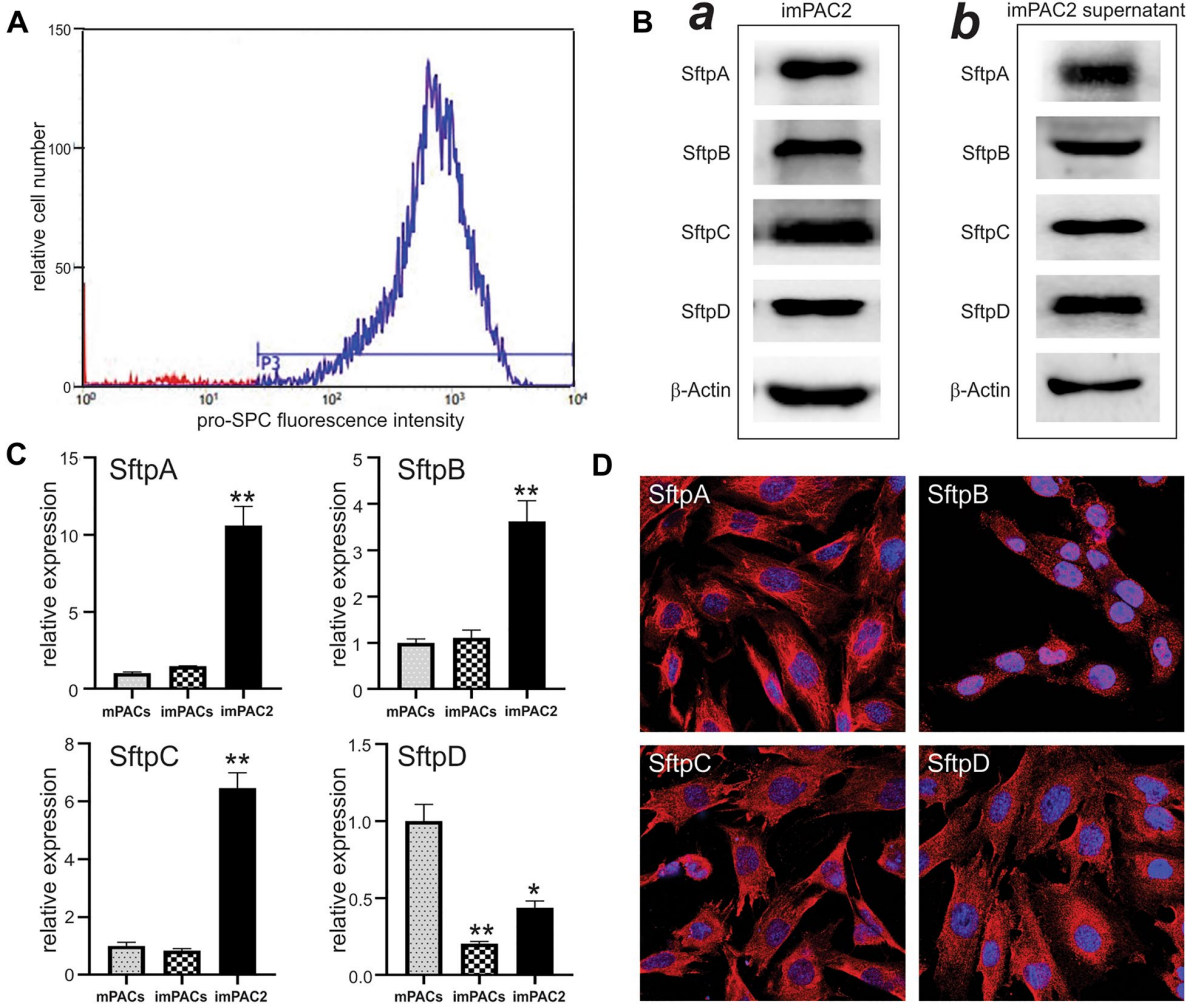
Selection and characteristics of the EpCAM^+^ cells of immortalized mouse alveolar type 2 cells (imPAC2). **A** FACS analysis of the isolated imPAC2 cells. The pooled imPACs were subjected to FACS sorting analysis with an EpCAM antibody; and the EpCAM^+^ cells were enriched with streptavidin-coated microbeads (Miltenyi Biotec), resulting in the imPAC2 cells (Additional file 1: Figure S1D), which were further confirmed for the presence of the AT2 marker pro-SPC with > 97% positivity. **B** Western blotting analysis of SftpA, SftpB, SftpC and SftpD expression in imPAC2 cells. The imPAC2 cells (***a***) and imPAC2 culture supernatant (***b***) were subjected to Western blotting using SftpA, SftpB, SftpC and SftpD antibodies, as well as long with β-actin antibody for internal loading control. **C** The qPCR-based expression analysis of AT2 markers SftpA, SftpB SftpC and SftpD expression in imPAC2 cells. Total RNA was isolated from subconfluent primary mPAC cells, imPACs and imPAC2 cells; and subjected to RT-qPCR analysis using gene-specific qPCR primers for AT2 makers *SftpA*, *SftpB*, *SftpC* and *SftpD*. *Gapdh* was used as a reference gene to normalize the cDNA levels among the samples. “**” p < 0.01, “*” p < 0.05, compared with that of the mPACs group. **D** Immunofluorescence analysis of SftpA, SftpB, SftpC and SftpD expression in imPAC2 cells. Subconfluent imPAC2 cells were subjected to immunostaining with primary antibodies against SftpA, SftpB, SftpC, SftpD. Cell nuclei were counterstained with DAPI. Representative images are shown. Staining without primary antibodies was used as a negative control (data not shown)

We sought to isolate the AT2 subpopulation from the imPACs pool by magnetic cell sorting (MACS) sorting of the EpCAM^+^ population as previously described [42]. As shown in the Additional file 1: Figure S1D, 54.9% of the immortalized imPACs pool were positive for the AT2 marker EpCAM, indicating that the vast majority of the recovered cells were alveolar epithelium cells. These results are highly close to the anticipated results since AT2 cells account for approximately 60% of alveolar epithelial cells. Nonetheless, since EpCAM is a pan-epithelial cell marker, we further validated the isolated EpCAM^+^ cells with the AT2-specific marker pro-SPC, and found that > 97% of the EpCAM^+^ cells were positive for pro-SPC (Fig. 3A). Western blotting analysis confirmed the expression of the AT2 surfactant proteins SftpA, SftpB, SftpC, and SftpD in both cell lysate (Fig. 3B-a) and the supernatants of the cultured enriched cells (Fig. 3B-b). We also carried out qPCR analysis and compared the relative expression levels of AT2 surfactant genes in the primary PACs (mPACs), the immortalized mPACs pool (imPACs), and EpCAM^+^ AT2 cells sorted from imPACs (imPAC2). Our results showed that the expression of *SftpA, SftpB*, and SftpC was significantly enriched in imPAC2 cells, compared with that in mPACs and imPACs (p < 0.01), while SftpD was highly expressed in the primary mPACs (Fig. 3C). Furthermore, immunofluorescence staining revealed that the enriched EpCAM^+^ cells were stained positive for the four surfactant proteins (Fig. 3D). Collectively, these results demonstrate that the enriched cells are representative of AT2 cells, and thus designated as imPAC2 cells.

### The imPAC2 cells form alveolar organoids more effectively than the imPACs

**Fig. 4:**
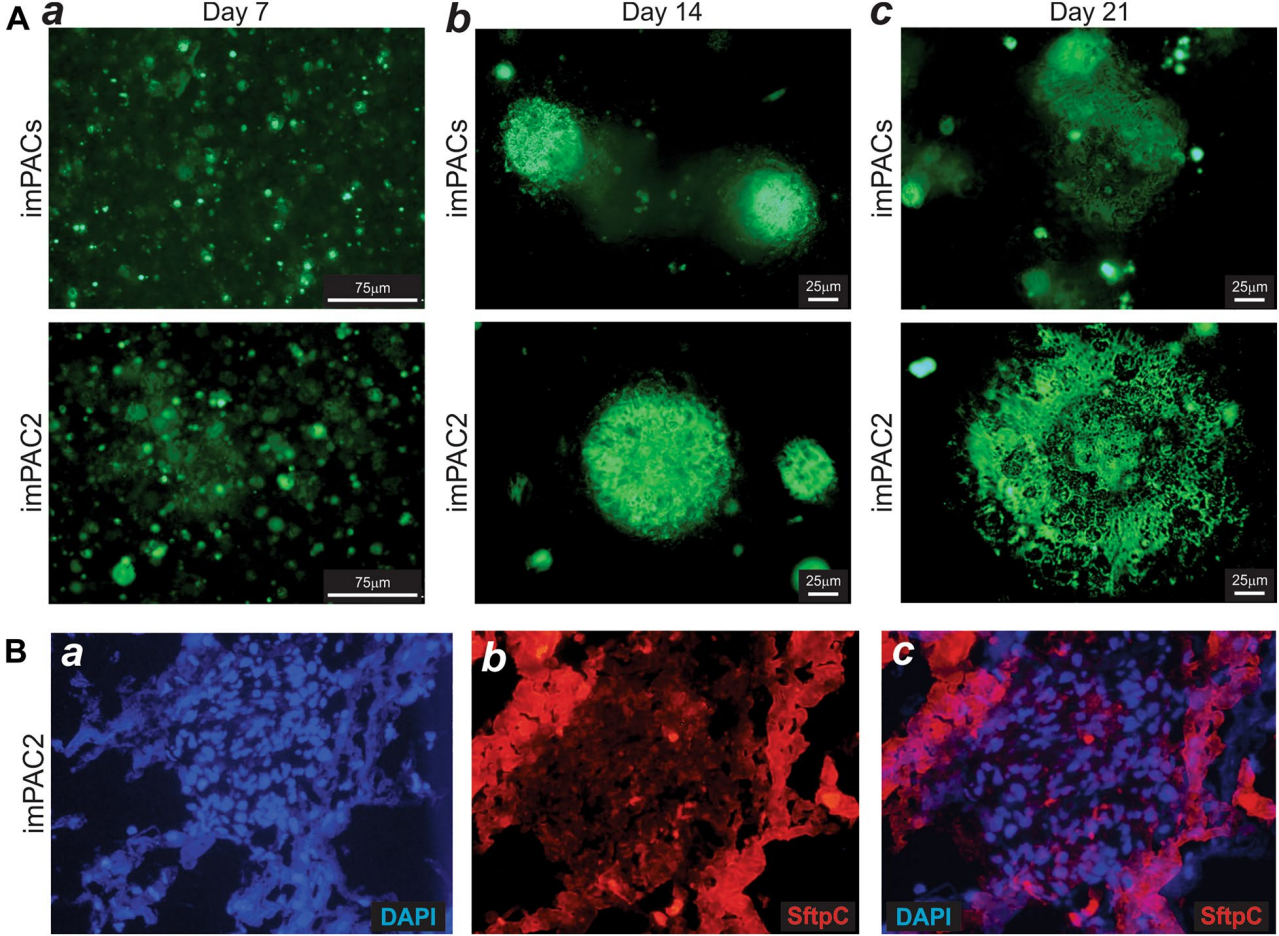
Alveolar organoid formation from imPACs and imPAC2 cells. **A** Subconfluent imPACs and imPAC2 cells were infected with Ad-GFP for 16 h and collected for organoid formation in Matrigel as described in the Methods. Sphere formation was recorded under a fluorescence microscope at day 7 (***a***), day 14 (***b***), and day 21 (***c***). Representative images are shown. **B** SftpC expression in the organoids. The 21-day organoids were harvested for whole mount immunefluorescent staining with the primary antibody against SftpC (red). Cell nuclei were counterstained with DAPI. Representative images are shown. Staining without primary antibodies was used as a negative control (data not shown)

We next tested whether the imPACs and imPAC2 cells could form alveolar organoids in culture. In order to easily track organoid formation, we first labeled the imPACs and imPAC2 cells by infecting the cells with Ad-GFP for 16 h. The GFP-labeled cells were collected, mixed with Matrigel, and seeded onto ultralow adhesive cell culture plates. Small spheroid structures were formed in both imPACs and imPAC2 cells at day 7 (Fig. 4A-a). However, significantly larger spheroids were observed in the imPAC2 cell group, compared with that in the imPACs group at day 14 and day 21 (Fig. 4A-bc). The spheroids formed from the imPAC2 cells also displayed more pocket-like or alveolar sacs-like structures than that from the imPACs at day 21 (Fig. 4A-c). Immunofluorescence staining revealed that most of cells in the 21-day organoids derived from the imPAC2 cells were stained positive for SftpC (Fig. 4B-abc). It is noteworthy that we did not detect the expression of AT1-specific markers Aqp5 and Rage in the imPAC2-derived organoids (data not shown). Taken together, these results suggest that, while both imPACs and imPAC2 cells can form spheroid structures, the imPAC2 cells may be a superior source to form 3D alveolar organoids and capable of self-renewal and undergoing terminal differentiation since the imPAC2 cells contain > 97% AT2 cells, compared with 54.9% in the imPACs.

### Silencing endogenous β-catenin significantly decreases the expression levels of AT2 markers in the imPAC2 cells

**Fig. 5:**
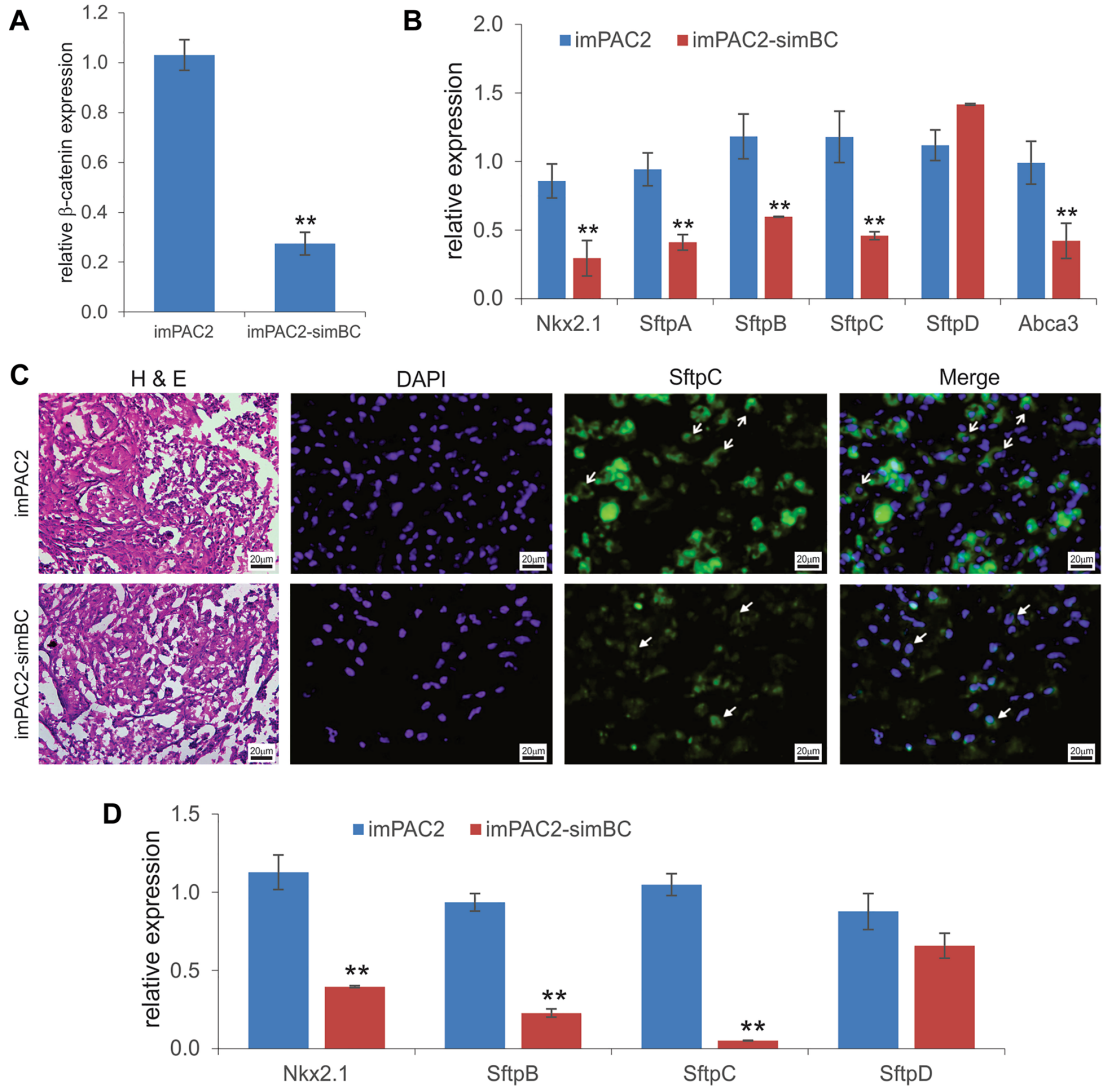
The essential role of β-catenin in maintaining the expression of alveolar type 2 (AT2) markers. **A** A retroviral vector expressing three siRNAs against mouse β-catenin was stably introduced into imPAC2 cells to generate the imPAC2-simBC cells. The qPCR analysis confirmed that β-catenin was effectively silenced in the imPAC2-simBC cells, compared with that in the imPAC2 cells (** p < 0.01). **B** Silencing β-catenin leads to the decreased expression of AT2 markers in vitro. Total RNA was isolated from subconfluent imPAC2 and imPAC2-simBC cells and subjected to qPCR analysis of the expression of *Nkx2.1*, *SftpA*, *SftpB*, *SftpC*, *SftpD*, and *Abca3*. “**” p < 0.01, compared with that of the imPAC2 group. **C** & **D** Silencing β-catenin leads to the decreased expression of AT2 markers in vivo. Subconfluent imPAC2 and imPAC2-simBC cells were collected, resuspended in scaffold material PPCNg/PBS, and subcutaneously injected into the flanks of athymic nude mice (5 × 10^6^ cells per injection). At 30 days after implantation, the subcutaneous masses were retrieved from the injection sites, fixed with paraformaldehyde, and subjected to H & E staining, and immunofluorescence staining with anti-SftpC antibody (**C**). Cell nuclei were counterstained with DAPI. Staining without primary antibodies was used as a negative control (not shown). Representative images are shown (**C**). Total RNA was also isolated from the freshly retrieved masses and subjected to qPCR analysis of the expression of *Nkx2.1*, *SftpB*, *SftpC*, and *SftpD* (**D**). *Gapdh* was used as a reference gene. “**” p < 0.01, compared with that of the imPAC2 cell group

It has been well established that Wnt/β-catenin signaling plays an important role in lung development, regeneration, and disease progression and that the Wnt-responsive AT2 cells play a critical role in lung alveologenesis [11, 13, 75]. We constructed a retroviral vector that expresses multiplex siRNAs targeting the coding region of mouse β-catenin and stably transduced the siRNAs into the imPAC2 cells to generate the imPAC2-simBC cells. TqPCR analysis revealed that β-catenin expression was significantly silenced in the imPAC2-simBC cells, compared with that of the imPAC2 cells (p < 0.01) (Fig. 5A). Moreover, the expression of the AT2 markers *Nkx2.1*, *SftpA, SftpB, SftpC,* and *Abca3* (except *SftpD*) drastically decreased in the imPAC2-simBC cells, compared with that of the imPAC2 cells (p < 0.01) (Fig. 5B), suggesting that Wnt/β-catenin signaling may be essential for maintaining the phenotype of AT2 cells.

To assess the effect of β-catenin silencing on the expression of AT2-related markers under in vivo 3D environment, we employed the thermoresponsive biocompatible PPCNg as both cell delivery vehicle and scaffold material since PPCNg was in liquid phase below 26 °C and solidified above 30 °C as previously described [52, 72]. When the PPCNg/cell mixes were subcutaneously implanted into the flanks of athymic nude mice, we found the both imPAC2-simBC/PPCNg group and imPAC2/PPCNg control group formed detectable masses at the injection sites at 30 days of injection without discernable difference in the sizes of masses, suggesting that the PPCNg scaffold may provide a 3D environment beneficial for the cell survival. Accordingly, histologic evaluation revealed that viable cells were presented in the retrieved masses, and that the SftpC expression was readily detected in the retrieved masses from the imPAC2 control, but not the imPAC2-simBC group (Fig. 5C). TqPCR analysis of the retrieved samples showed that the expression of the AT2-related markers *Nkx2.1*, *SftpB, SftpC,* and *Abca3* (except *SftpD*) drastically decreased in the imPAC2-simBC group, compared with that of the imPAC2 control group (p < 0.01) (Fig. 5D). Collectively, these results strongly suggest that Wnt/β-catenin may play an important role in maintaining the phenotype and marker expression of the pulmonary AT2 cells.

### TGF-β1 induces alveolar epithelial fibrosis-like response by regulating the expression of epithelial-mesenchymal transition (EMT) markers in the imPAC2 cells

Idiopathic pulmonary fibrosis (IPF) is a devastating disease manifested by progressive and severe scar formation in the alveolar regions of the lung [5, 76]. While the exact mechanism underlying IPF pathogenesis remains to be fully understood, it is hypothesized that cross talk between alveolar epithelium and its associated mesenchyme may be dysregulated, leading to the uncontrolled proliferation of extracellular matrix-producing cells [76]. Increasing evidence indicates that depletion or senescence of AT2 cells drives pulmonary fibrosis [5, 76–78]. Furthermore, TGF-β1 is considered a key initiating factor for pulmonary fibrosis [79, 80].

**Fig. 6:**
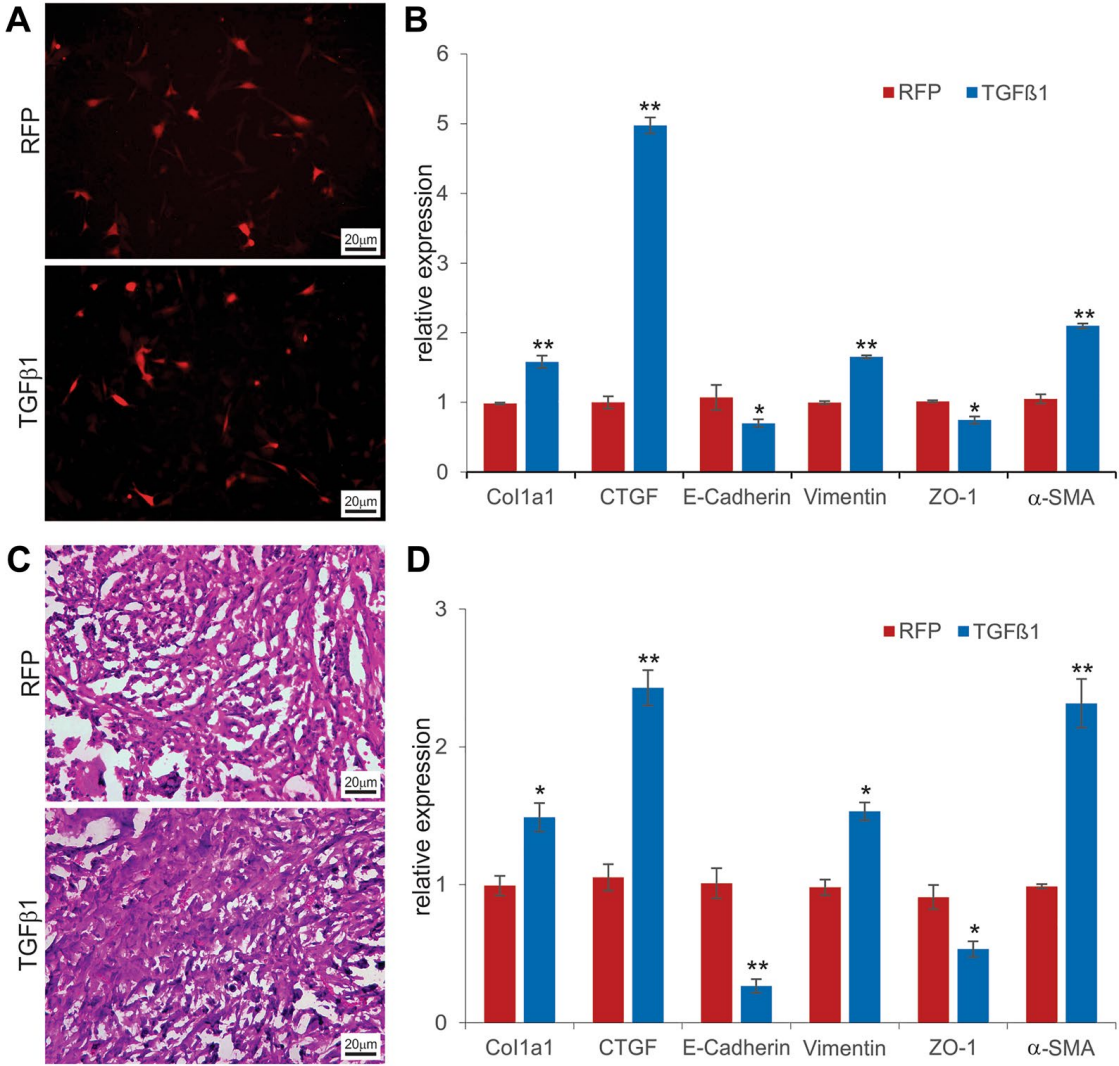
TGFβ1-induced expression of pulmonary fibrosis-related markers in imPAC2 cells. **A** & **B** Subconfluent imPAC2 cells were infected with Ad-RFP or Ad-TGFβ1 (**A**). At 72 h after infection, total RNA was isolated from the infected cells and subjected to qPCR analysis of the expression of collagen I (Col1a1), CTGF, E-cadherin, vimentin, zonula occluden-1 (ZO-1), and α-SMA (**B**). **C** & **D** Subconfluent imPAC2 cells were infected with Ad-TGFβ1 or Ad-RFP for 16 h. The infected cells were collected and mixed with PPCNg/PBS and injected into the flanks of athymic nude mice subcutaneously. At 30 days after implantation, the subcutaneous masses were retrieved from the injection sites, fixed with paraformaldehyde, and subjected to H & E staining (**C**). Total RNA was also isolated from the freshly retrieved masses and subjecte d to qPCR analysis of the expression of Col1a1, CTGF, E-cadherin, vimentin, ZO-1, and α-SMA (**D**). *Gapdh* was used as the reference gene. “**” p < 0.01, “*” p < 0.05, compared with that of the Ad-RFP infection group

We sought to test how the imPAC2 cells would respond to TGF-β1. The imPAC2 cells were infected with Ad-TGF-β1 or Ad-RFP with the same infection efficiency (Fig. 6A), and the adenovirus-mediated expression of TGF-β1 was further confirmed (Additional file 1: Figure S2A). TqPCR analysis revealed that, upon TGF-β1 stimulation, the expression of α-SMA, vimentin, CTGF, and Col1a1 were significantly upregulated, while the expression of E-cadherin and ZO-1 was downregulated in the imPAC2 cells (Fig. 6B). Furthermore, the Ad-TGF-β1 or Ad-RFP infected imPAC2 cells were mixed with PPCNg scaffold and subcutaneously implanted into the flanks of athymic nude mice for 30 days. Histologic evaluation of the retrieved samples indicated that the Ad-TGF-β1-transduced imPAC2 group were more cellular with collagen clustered and matrix accumulation regions, compared with that of the Ad-RFP infection group (Fig. 6C). Further TqPCR analysis of the retrieved samples showed that α-SMA, vimentin, CTGF, and Col1a1 were significantly upregulated, while E-cadherin and ZO-1 were downregulated in the Ad-TGF-β1-transduced imPAC2 group, compared with that of the Ad-RFP control group (Fig. 6D). Taken together, these results demonstrated that TGF-β1 stimulation resulted in an EMT-like response in the imPAC2 cells, suggesting that TGF-β1-induced EMT of AT2 cells may in part contribute to the pathogenesis of pulmonary fibrosis.

### Introduction of oncogenic *KRAS* and mutant *TP53* in the imPAC2 cells induces tumor-like phenotype and activates lung cancer-associated pathways

**Fig. 7:**
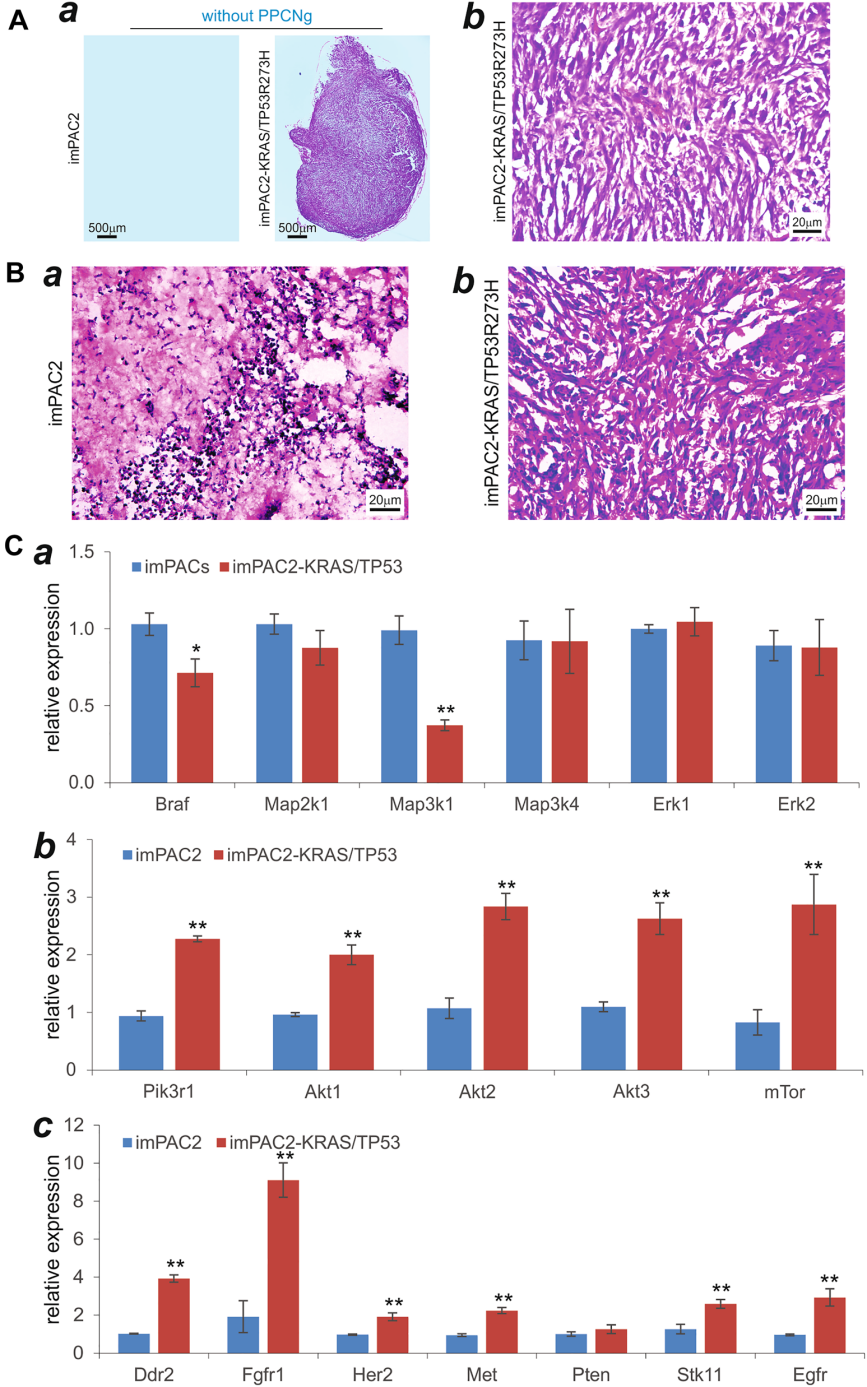
The activation of tumorigenic pathways by overexpressing oncogenic *KRAS* and mutant *TP53* in the imPAC2 cells. Retroviral vectors expressing oncogenic human KRAS and mutant TP53-R273H were stably transduced into imPAC2 cells, resulting in the imPAC2-KRAS/TP53R273H cell line. The expression of KRAS and TP53R273H was confirmed by qPCR (Additional file 1: Figure S2B). Subconfluent imPAC2 and imPAC2-KRAS/TP53R273H cells were harvested, resuspended in PBS alone or PPCNg/PBS and subcutaneously injected into the flanks of athymic nude mice. At 30 days after injection, in the absence of PPCNg scaffold subcutaneous masses were only found in the imPAC2-KRAS/TP53R273H injection group, but not in the imPAC2 group (**A-a**), followed by H & E histologic analysis (**A-b**). Subcutaneous masses were retrieved from both imPAC2-KRAS/TP53R273H injection group and the imPAC2 group, and subjected to H & E staining (**B-ab**). Representative results are shown. Total RNA was also isolated from the retrieved masses in the PPCNg groups and subjected to qPCR analysis of the expression of RAS-MAK pathway genes (**C-a**), PI3K/AKT/mTOR pathway genes (**C-b**), and lung cancer-associated genes (**C–c**). *Gapdh* was used as the reference gene. “**” p < 0.01, “*” p < 0.05, compared with that of the imPAC2 control group

It has been well established that AT2 cells as the most common cell of origin for lung adenocarcinoma [81–83]. Like many other solid tumors, *KRAS* and *TP53* are frequently mutated in lung cancer [83]. Here, we sought to text the biological consequences when mutant *KRAS* and *TP53* were introduced into the imPAC2 cells. By constructing retroviral vector expressing the full-length coding region of human KRAS G12C or TP53R273H, we co-infected the imPAC2 cells with the packaged retrovirus supernatants, and established the stable imPAC2-KRAS/TP53R273H line. The concurrent expression of oncogenic *KRAS* and mutant TP53 in this line was separately verified by TqPCR analysis (Additional file 1: Figure S2B-ab). The imPAC2-KRAS/TP53R273H cells were mixed with or without the scaffold PPCNg and injected subcutaneously into the flanks of athymic nude mice for 30 days. Consistent with their non-tumorigenic feature, the imPAC2 cells failed to form any subcutaneous masses in the absence of PPCNg scaffold, while the imPAC2-KRAS/TP53R273H cells formed obvious masses without PPCNg scaffold (Fig. 7A-a), and exhibited a highly proliferative feature histologically (Fig. 7A-b), suggesting that concurrent overexpression of oncogenic *KRAS* and mutant *TP53* may be sufficient to render the imPAC2 cells tumor-like phenotype.

Accordingly, histologic evaluation of the retrieved samples mixed with PPCNg indicated that the imPAC2-KRAS/TP53R273H group exhibited higher cellularity with many mitotic cells, compared with that of the imPAC2 control group (Fig. 7B a* vs. b*). The retrieved samples from the PPCNg groups were further subjected to TqPCR analysis of the expression of the downstream pathways regulated by oncogenic *KRAS* and/or mutant *TP53*, as well as lung cancer-related genes. We found that while *Braf* and *Map3k1* were downregulated, the expression of most members of the MAPK pathway was not significantly affected by oncogenic *KRAS* and mutant *TP53* in the imPAC2 cells (Fig. 7C-a). However, stable expression of oncogenic *KRAS* and mutant *TP53* in the imPAC2-KRAS/TP53R273H cells significantly upregulated the expression of the members of the PI3K/AKT/mTOR pathway such as *Pik3r1, Akt1, Akt2, Akt3,* and *mTor* (Fig. 7C-b). Lastly, the expression of lung cancer-related genes including *Ddr2, Fgfr1, Her2, Met, Stk11,* and *Egfr* was also significantly upregulated in the imPAC2-KRAS/TP53R273H group, compared with that of the imPAC2 control group (Fig. 7C-c). Interestingly, the *Pten* expression was not significantly affected by oncogenic *KRAS* and mutant *TP53* although the *Pten* basal expression level was extremely low in the imPAC2 cells. Collectively, these results demonstrated that concurrent expression of oncogenic *KRAS* and mutant *TP53* in the imPAC2 cells rendered the imPAC2 cells tumor-like phenotype and upregulated numerous lung cancer-related pathways, suggesting the imPAC cells may be exploited to model lung tumorigenesis.

## Discussion

As the main cell type of the alveolar epithelial cells, AT2 cells are heterogeneous and function as progenitor cells with self-renewal capability and differentiation potential into AT1 cells for lung repair after injury [3, 5–10]. Despite the pivotal role of alveoli and AT2 cells in lung function and disease, the lack of clinically relevant AT2-based cell models hampers our understanding about lung biology and pathogenesis of many pulmonary diseases. Here, we developed the reversibly immortalized mouse pulmonary alveolar type 2 cells (imPAC2) and analyzed their potential in forming alveolar organoids for modeling pulmonary diseases.

Here, we isolated primary mouse pulmonary alveolar cells (mPACs) from CD1 newborn pups and immortalized the primary cells with a retroviral vector expressing SV40 LTA that is flanked with FRT sites. The immortalized cell pool imPACs maintained long-term proliferative capability, which could be reversed by FLP-mediated removal of SV40 LTA. Xenograft experiments showed that the imPACs were non-tumorigenic, consistent with what we previously found that SV40 LTA-immortalized primary progenitor cells from various tissues are in general non-tumorigenic [26–37, 84–86]. It is noteworthy that, while imPACs do not form any subcutaneous masses, the addition of the PPCNg biocompatible scaffold can sustain the cell survival in 3D microenvironment. By FACS sorting for the EpCAM^+^ cells from the imPACs, we further isolated the AT2-enriched subpopulation (imPAC2) cells, which were shown to express AT2 markers and were able to form alveolar organoids. Thus, unlike many of the currently used lung epithelial cell lines, such as human lung cancer (e.g., A549 and H441), mouse SV40 Large T transgenic lung tumors (e.g., MLE12 and MLE15), and spontaneously immortalized Golden hamster (e.g., M3E3/C3) cell lines, many of which do not always recapitulate the morphological, functional, and/or phenotype of the AT2 cells [17], we believe the imPAC2 cells are a genuine representative of AT2 cells.

The use of organoids to model lung development and diseases has gained significant attention recently [87–89]. Currently, lung organoids are usually constructed from primary tissue, lung progenitors, or induced pluripotent stem cells (iPSCs) in specific 3D culture conditions [87–91]. The common methods for isolating primary AT2 cells for organoid culture are using FACS or MACS with a unique antibody, and the generation of 3D organoids usually requires the use of a cell support that mimics it the physiological environment from mesenchymal cells, endothelial cells, and/or Pdgfra + fibroblasts [8, 92, 93]. It was reported that human iPSCs could be used to differentiate towards alveolar epithelial cells, such as AT2 cells and lung organoids, as a possible in vitro model for investigating lung diseases and drug screening [94, 95]. However, maintenance of primary alveolar epithelial cells under 3D conditions and/or replication of the lung alveolus in vitro remain a challenge. Furthermore, the induction of iPSCs towards alveolar epithelial lineage and/or AT2 phenotype is complex, time-consuming, empirical and finicky. Here, we demonstrated that the imPAC2 cells effectively formed organoid-like structures that were SftpC^+^ although further investigation is warranted to determine whether imPAC2 cells can differentiate into AT1 cells.

We also demonstrated that silencing endogenous catenin significantly decreased the expression of AT2 markers in the imPAC2 cells, suggesting that canonical Wnt/β-catenin may play important role in maintaining AT2 phenotype. In fact, it has been shown that Wnt signaling was critical in maintaining AT2 stemness and regulating the differentiation of AT2 progenitors to AT1 cells [11–13]. The activation of Wnt signaling in a rare subpopulation of AT2 was shown to block the differentiation of AT2 to AT1 cells after epithelial injury [12]. It was also reported that β-catenin was required to maintain the lung progenitor during fetal development in mice [96]. Nonetheless, the process of AT2-to-AT1 differentiation is likely complex and involves several signaling pathways including Wnt/β-catenin, Notch, and BMP/SMAD [13, 97, 98]. Thus, it is conceivable that the imPAC2 cells can be used a valuable cell model to delineate mechanism(s) underlying the AT2-to-AT1 differentiation process.

When the imPAC2 cells were treated with TGF-β1, we found that TGF-β1 induced a fibrosis-like response by regulating the expression of EMT markers both in vitro and in vivo. Idiopathic pulmonary fibrosis (IPF) is a devastating disease manifested by progressive and severe scar formation in the alveolar regions of the lung [5, 76]. Increasing evidence indicates that depletion or senescence of AT2 cells drives pulmonary fibrosis [5, 76–78]. TGF-β1 is considered a key initiating factor for pulmonary fibrosis [79, 80]. Our results demonstrated that TGF-β1 stimulation resulted in an EMT-like response in the imPAC2 cells, suggesting that TGF-β1-induced EMT of AT2 cells may in part contribute to the pathogenesis of pulmonary fibrosis. Interestingly, an earlier study employed a surfactant protein knock-in mouse model and found that, upon bleomycin treatment, multiple stromal populations contributing to pulmonary fibrosis without evidence for epithelial to mesenchymal transition as labeled epithelial cells generated AT1 and AT2 cells but not fibroblasts [99]. While such in vivo findings are seemingly convincing, bleomycin-induced lung injury may be fundamentally different from TGF-β1-induced pulmonary fibrosis. Furthermore, cell-type specific promoter-driven cell tracing studies may be problematic if the labeled cells become different cell types which may lose the expression of the tracers. Nonetheless, it would be interesting to investigate whether or not TGF-β1-induced EMT of AT2 cells contributes to the pathogenesis of pulmonary fibrosis.

Lastly, we demonstrated that concurrent expression of oncogenic *KRAS* and mutant *TP53* in the imPAC2 cells induced a tumor-like phenotype and activated lung cancer-associated pathways. It has been well established that AT2 cells as the most common cell of origin for lung adenocarcinoma [81–83]. Like many other solid tumors, *KRAS* and *TP53* are frequently mutated in lung cancer [83]. We found the concurrent expression of oncogenic *KRAS* and mutant *TP53* in the imPAC2 cells led to the formation of subcutaneous masses with high proliferative activity even in the absence of the 3D PPCNg scaffold, and upregulated the expression of the members of the PI3K/AKT/mTOR pathway and lung cancer-related genes in vivo. Our results suggest that concurrent overexpression of oncogenic *KRAS* and mutant *TP53* may be sufficient to render the imPAC2 cells tumor-like phenotype so that the imPAC cells may be exploited to model lung tumorigenesis.

## Conclusions

We immortalized mouse pulmonary alveolar type 2 cells (imPAC2) with SV40 LTA that is flanked with FRT sites. The imPACs were non-tumorigenic, and maintained long-term proliferative activity that was reversible by FLP-mediated removal of SV40 LTA. AT2-enriched subpopulation (imPAC2) was obtained by FACS sorting for the EpCAM^+^ cells from the imPACs, and was shown to express AT2 markers and form alveolar organoids. Silencing β-catenin decreased the expression of AT2 markers in the imPAC2 cells, while TGF-β1 induced alveolar fibrosis-like response by regulating the expression of EMT markers in the imPAC2 cells. Concurrent expression of oncogenic *KRAS* and mutant *TP53* in the imPAC2 cells induced a tumor-like phenotype and activated lung cancer-associated pathways. Collectively, our results strongly suggest that the imPAC2 may represent a genuine population of AT2 cells that can be further explored to form AT2-based alveolar organoids for modeling pulmonary diseases.

## Supplementary Information

**Additional file 1: Table S1** List of Oligonucleotides Used in the Study. **Figure S1.** Expression of more alveolar markers in imPACs. **Figure S2**. TqPCR analysis of exogenous gene expression.

## Data Availability

All data generated or analyzed during this study are included in this published article and its supplemental materials.
