# Supplementary material for: Modeling lung diseases using reversibly immortalized mouse pulmonary alveolar type 2 cells (imPAC2)
Source: Cell Biosci. 2022 Sep 22;12:159. doi: 10.1186/s13578-022-00894-4 (PMC9502644; doi:10.1186/s13578-022-00894-4)
Supplement: Supplementary file 1 — Additional file 1: Table S1 List of Oligonucleotides Used in the Study. Figure S1. Expression of more alveolar markers in imPACs. Figure S2. TqPCR analysis of exogenous gene expression. [file 13578_2022_894_MOESM1_ESM.docx]

**Modeling lung diseases using reversibly immortalized mouse pulmonary alveolar type 2 cells (imPAC2)**

**Running Title: Modeling lung diseases using the imPAC2 cells**

Linghuan Zhang^1,2^, Wenping Luo^3,2^, Jiang Liu^1^, Maozhu Xu^1^, Qi Peng^4^, Wenjing Zou^1^, Jingyi You^1^, Yi Shu^1,2^, Piao Zhao^2,5^, William Wagstaff^2^, Guozhi Zhao^2,5^, Kevin Qin^2,6^, , Rex C. Haydon^2^, Hue H. Luu^2^, Russell R. Reid^2,7,8^, Yang Bi^1,2^, Tianyu Zhao^9^,Tong-Chuan He^2,7^*, and Zhou Fu^1^*

^1^ Stem Cell Biology and Therapy Laboratory, Ministry of Education Key Laboratory of Child Development and Disorders, and the Department of Respiratory Diseases, The Children's Hospital of Chongqing Medical University, Chongqing 400014, China

^2^ Molecular Oncology Laboratory, Department of Orthopaedic Surgery and Rehabilitation Medicine, The University of Chicago Medical Center, Chicago, IL 60637, USA

^3^ Laboratory Animal Center, Southwest University, Chongqing 400715, China

^4^ University-Town Hospital, Chongqing Medical University, Chongqing 401331, China

^5^ Departments of Orthopaedic Surgery and Urology, The First Affiliated Hospital of Chongqing Medical University, Chongqing 400046, China

^6^ Rosalind Franklin University of Medicine, North Chicago, IL 60064, USA

^7^  Department of Surgery, The University of Chicago Medical Center, Chicago, IL 60637, USA

^8^  Laboratory of Craniofacial Suture Biology and Development, Department of Surgery Section of Plastic Surgery, The University of Chicago Medical Center, Chicago, IL 60637, USA

^9^ Chongqing Key Laboratory of Oral Diseases and Biomedical Sciences, and the Stomatological Hospital of Chongqing Medical University, Chongqing 401147, China

* Corresponding authors

**CORRESPONDENCES**

T.-C. He, MD, PhD

Molecular Oncology Laboratory

The University of Chicago Medical Center

5841 South Maryland Avenue, MC3079

Chicago, IL 60637, USA

Tel. (773) 702-7169

Fax: (773) 834-4598

E-mail: [tche@uchicago.edu](mailto:tche@uchicago.edu)

Zhou Fu, MD

Department of Respiratory Medicine

The Children’s Hospital

Chongqing Medical University

No. 136, Zhong Shan 2nd Road, Yuzhong District

Chongqing 400014, China

Tel.: +86 13983021280

Email: [fu_zhou79@126.com](mailto:fu_zhou79@126.com)

**Supporting Materials**

**Table S1**: List of Oligonucleotides Used in the Study.

| **Table S1. Oligonucleotides Used in the Study** | | | |
| --- | --- | --- | --- |
| Species | Gene | Sequence | Use |
| SV40 | *SV40 T antigen* | AGAATGGATGGCTGGAGTTGCT | qPCR |
|  |  | AGCTCAAAGTTCAGCCTGTCCA |  |
| mouse | *Aqp5* | GACCTGTGAGTGGTGGCC |  |
|  |  | TGGCGCCAACCAGTACAG |  |
|  | *Ager* | GGTCACAGAAACCGGCGA |  |
|  |  | GGGGCCTTCCTCTCCTCA |  |
|  | *Pdpn* | AAATGCCGACTGTGCCGA |  |
|  |  | GGGAAGAGCTCGGGAGGA |  |
|  | *SftpA1* | TGCGACCATGACCCACAC |  |
|  |  | GTGGGCAGAGCACAGGAG |  |
|  | *SftpB* | GACAAGCCTCAGCCTGCA |  |
|  |  | GGGGGAGCCAGAAACCAC |  |
|  | *SftpC* | GCTGTGAGCACCCTGTGT |  |
|  |  | TTTCTGGGCAGGAGCAGC |  |
|  | *SftpD* | CCCCTGGTGTGCAAGGAG |  |
|  |  | GGAAGCCCGCTTTCACCT |  |
|  | *Abca3* | GTGCTGCCAAACCACTGC |  |
|  |  | CACAAACTTGCCCACGCC |  |
|  | *Ctsh* | GCCCCTACCCTTCCTCCA |  |
|  |  | GGGCACAATCCACCAGCT |  |
|  | *Lamp-1* | GGAGTCACGCCGGCTATC |  |
|  |  | GTGCCTCCCTTCCACACC |  |
|  | *Lamp-2* | CTGGCTACCATGGGGCTG |  |
|  |  | TGAGGTTGACAGCTGCCG |  |
|  | *Muc1* | GCCACCAGTCCAGACCAC |  |
|  |  | GCACCGAGGAGCCATTGT |  |
|  | *Nkx2.1* | CATGGGCAAGGGTCAGGG |  |
|  |  | GCCCTCCATGCCCACTTT |  |
|  | *Gapdh* | GCCTCGTCCCGTAGACAAAA |  |
|  |  | TTCCCATTCTCGGCCTTGAC |  |
|  | *Braf* | CTGCACACCCACCTCCTG |  |
|  |  | TCGAGCGCTCCTGACTCT |  |
|  | *Map2k1* | CGAACTGGGAGCTGGCAA |  |
|  |  | TACCTGCAGCTCCCGGAT |  |
|  | *Map3k1* | TCCGTCCAGAGAGCTGCT |  |
|  |  | TCTGGCTGTTCAGTGGCG |  |
|  | *Map3k4* | CTCGATGCCCATGCCGAT |  |
|  |  | TCCCCTCTGCTCCCTGTC |  |
|  | *Erk1* | CACGTTGGTACAGAGCTCCA |  |
|  |  | TGTGATTCAGCTGGTCAAGG |  |
|  | *Erk2* | TGCCCCTCACAGCAGTTT |  |
|  |  | GGCCTGCTTCCATTCAGA |  |
|  | *Pik3r1* | GCGAGACGGCACTTTCCT |  |
|  |  | TTGTGCTGCACGAGGGAG |  |
|  | *Akt1* | CCCTTCTACAACCAGGACCA |  |
|  |  | CATGATCTCCTTGGCATCCT |  |
|  | *Akt2* | GAAGACTGAGAGGCCACGAC |  |
|  |  | CTTGTAATCCATGGCGTCCT |  |
|  | *Akt3* | GAAACTGGCCACTTCTGCTC |  |
|  |  | ACTGAGGTGTGGTGGAGACC |  |
|  | *mTor* | TCATGCCTTTCCTGCGCA |  |
|  |  | CGGGTTTGGGTCAGGGTC |  |
|  | *Ddr2* | GGGCCTTTGGGGTGACTC |  |
|  |  | CAGAGTCGGGGCAAAGGG |  |
|  | *Fgfr1* | TTGGGGTCAACTTGGCAACT |  |
|  |  | CACAGCTGCCAAAACTGCAT |  |
|  | *Her2* | ACTGTCTGCCATGCCACC |  |
|  |  | CAGCGAGCCACACAGGAA |  |
|  | *Met* | CACGGCAGAAACCCCCAT |  |
|  |  | TGCTGCAGTCCCGACAAG |  |
|  | *Pten* | TCACCATTGCCAGGGCTG |  |
|  |  | TCACCATTGCCAGGGCTG |  |
|  | *Stk11* | ACAGTGTGCCGGAGAAGC |  |
|  |  | GCCCGGCTTGATGTCCTT |  |
|  | *Egfr* | CAGTGGGCAACCCTGAGTAT |  |
|  |  | GGGCCCTTAAATATGCCATT |  |
|  | *Col1a1* | GCTCCTCTTAGGGGCCACT |  |
|  |  | CCACGTCTCACCATTGGGG |  |
|  | *Ctgf* | AAGGACCGCACAGCAGTT |  |
|  |  | AACAGGCGCTCCACTCTG |  |
|  | *E-cadherin* | ACGAGGGCAGTGGTTCTG |  |
|  |  | CATGTCCGCCAGCTTCTT |  |
|  | *Vimentin* | CAGATGCGTGAGATGGAAGA |  |
|  |  | TCCAGCAGCTTCCTGTAGGT |  |
|  | *Zo-1* | CCCGAGACCTGGACTCCA |  |
|  |  | GTTCGAGGCAGCTGCTCA |  |
|  | *α-Sma* | CTGACAGAGGCACCACTGAA |  |
|  |  | CATCTCCAGAGTCCAGCACA |  |
| human | *KRAS* | TGTGGTAGTTGGAGCTGGTG |  |
|  |  | TGACCTGCTGTGTCGAGAAT |  |
|  | *TP53* | GGCCCACTTCACCGTACTAA |  |
|  |  | GTGGTTTCAAGGCCAGATGT |  |
| mouse | *β-catenin* | GCAGTTTGACGCTGCTCAT | siRNAs |
|  |  | CCAGGTGGTAGTTAATAAA |  |
|  |  | GCACCATGCAGAATACAAA |  |
| human | *KRAS G12C* | cgacTAGGGATAACAGGGTAATaccatgggaATGACTGAATATAAACTTGTGGTAG | cloning |
|  |  | gtacTAGGGATAACAGGGTAATTTACATTATAATGCATTTTTTAAT |  |
|  | *TP53-R273H* | cgacTAGGGATAACAGGGTAATaccaccATGGAGGAGCCGCAGTCAGATCCTAG |  |
|  |  | gtacTAGGGATAACAGGGTAATtcagtctgagtcaggcccttctgtc |  |


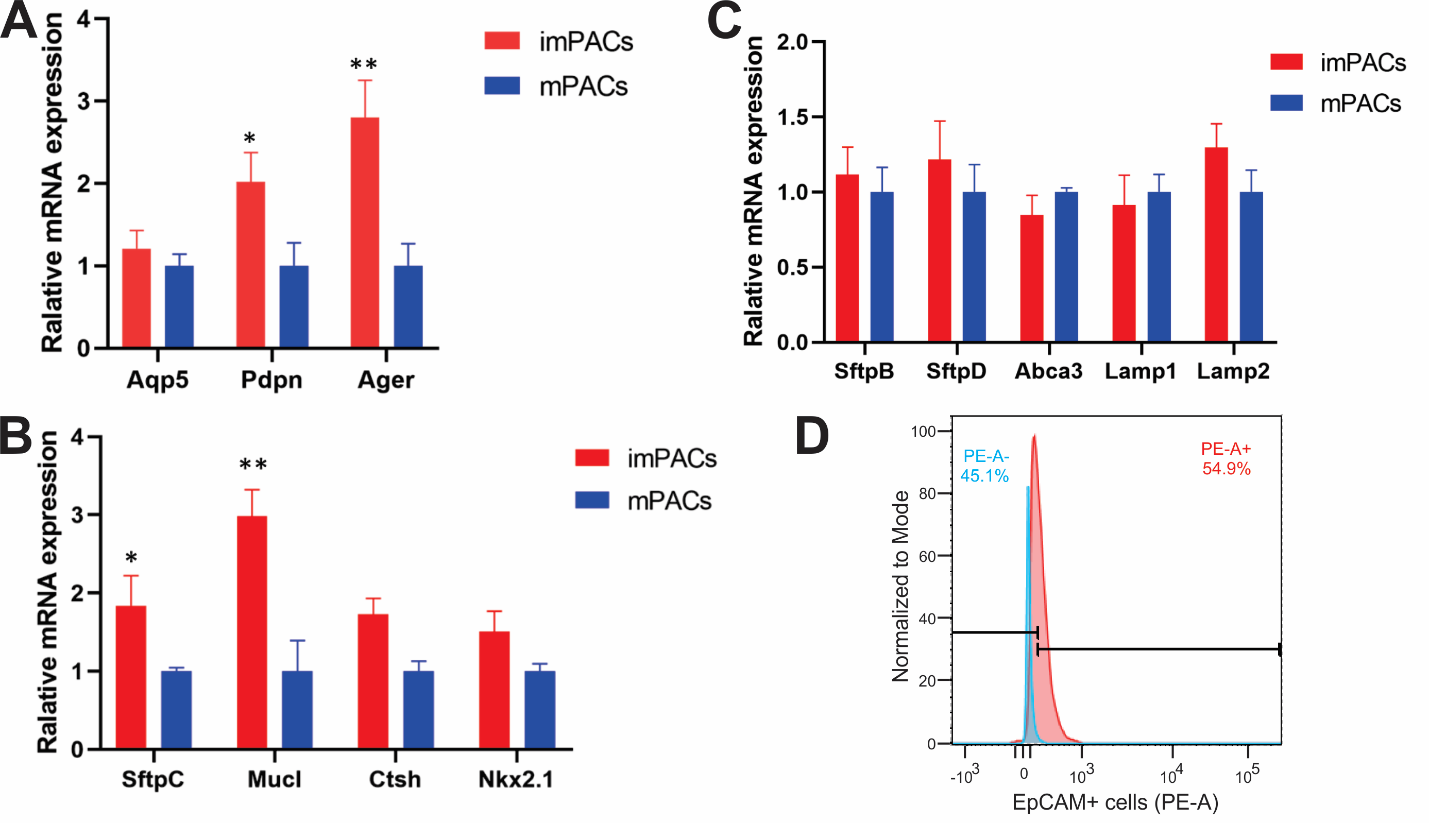


**Figure S1**. **Expression of more alveolar markers in imPACs**. Subconfluent mPACs and imPACs were cultured for 24h. Total RNA was isolated and subjected to qPCR analysis of AT1 and AT2 markers Aqp5, Pdpn, and Ager (**A**), SftpC, Muc1, Ctsh, and Nkx2.1 (**B**), SftpB, SftpD, Abca3, Lamp1, and Lamp2 (**C**). *Gapdh* was used as a reference gene. “**” p<0.01 compared with that of the mPACs group. (**D**) FACS analysis of the AT2 marker EpCAM+ cells in the imPACs pool. The imPACs were fixed, labeled with the EpCAM antibody and subjected to FACS analysis. The assay was done in triplicate, and the representative results are shown.


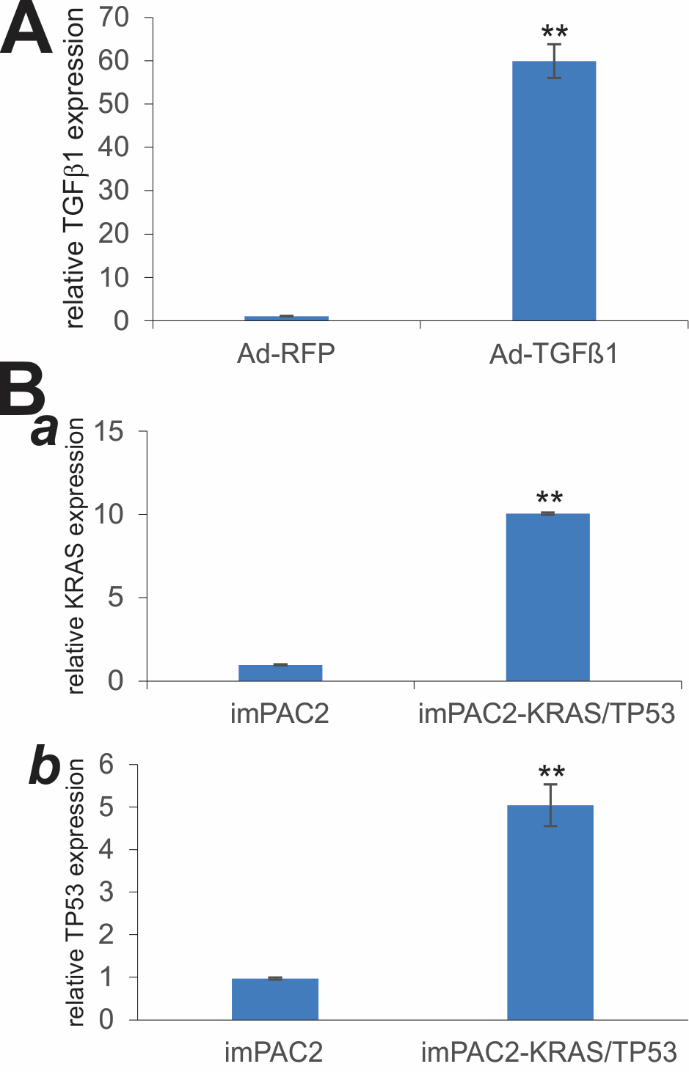


**Figure S2. TqPCR analysis of exogenous gene expression**. (**A**) Confirmation of adenovirus Ad-TGFβ1 mediated TGFβ1 expression. Subconfluent imPAC2 cells were infected with Ad-TGFβ1 or Ad-RFP. At 36h post infection, total RNA was isolated and subjected to qPCR analysis of TGFβ1 expression. *Gapdh* was used as a reference gene. “**” p<0.01 compared with that of the Ad-RFP group. (**B**) Subconfluent imPAC2 and imPAC2-KRAS/TP53 cells were cultured for 24h. Total RNA was isolated and subjected to qPCR analysis of KRAS (***a***) and TP53 (***b***) expression using primers for human KRAS and TP53, respectively. *Gapdh* was used as a reference gene. “**” p<0.01 compared with that of the imPAC2 group.
